# The End of Passive Collecting: The Role and Responsibility of Archivists in the COVID-19 Era

**DOI:** 10.1177/1550190620980839

**Published:** 2021-06

**Authors:** Elizabeth Mariano Mubarek

**Affiliations:** 1 Lexington Historical Society, MA, USA

**Keywords:** archives, subject focus, museum, collections, history, archivist, profession, COVID-19, rapid response collecting, contemporaneous collecting, collecting initiative

## Abstract

Set against the backdrop of the COVID-19 pandemic, this article argues that it is imperative that archivists be strongly attuned to current events, both locally and worldwide, so as to best serve their communities. Historic events should not necessarily be documented and recorded retroactively; rather, professional archivists have a responsibility to actively spearhead initiatives to collect contemporaneous documents, ephemera, and artifacts that record history as it is occurring, thus offering more personalized insights into the firsthand impact of significant events on daily life. Various institutions have already risen to this task and undertaken “rapid response collecting,” a relatively new method focused on immediate collecting during moments of extreme historical importance, which has gained considerable momentum throughout the course of the ongoing pandemic. Though the obstacles of collecting during a pandemic are numerous, as many archivists are working remotely or have limited access to physical collections, the benefits to future generations are invaluable.

## Introduction

“Throughout the U.S. the epidemic raged,” wrote Dr. Fred S. Piper. “Schools closed for about three weeks, and churches, theaters, lodges, etc. postponed meetings.”^1^

As of late March 2020, the above statement could be attributed to nearly any resident of any town in the United States, as the nation was experiencing the early effects of COVID-19. Seemingly overnight, what many considered “normal life” was altered drastically in a way that no one could have foreseen. Schools shut down, businesses closed, events were cancelled, vacations were postponed, and life was put on hold indefinitely.

**Figure 1.**
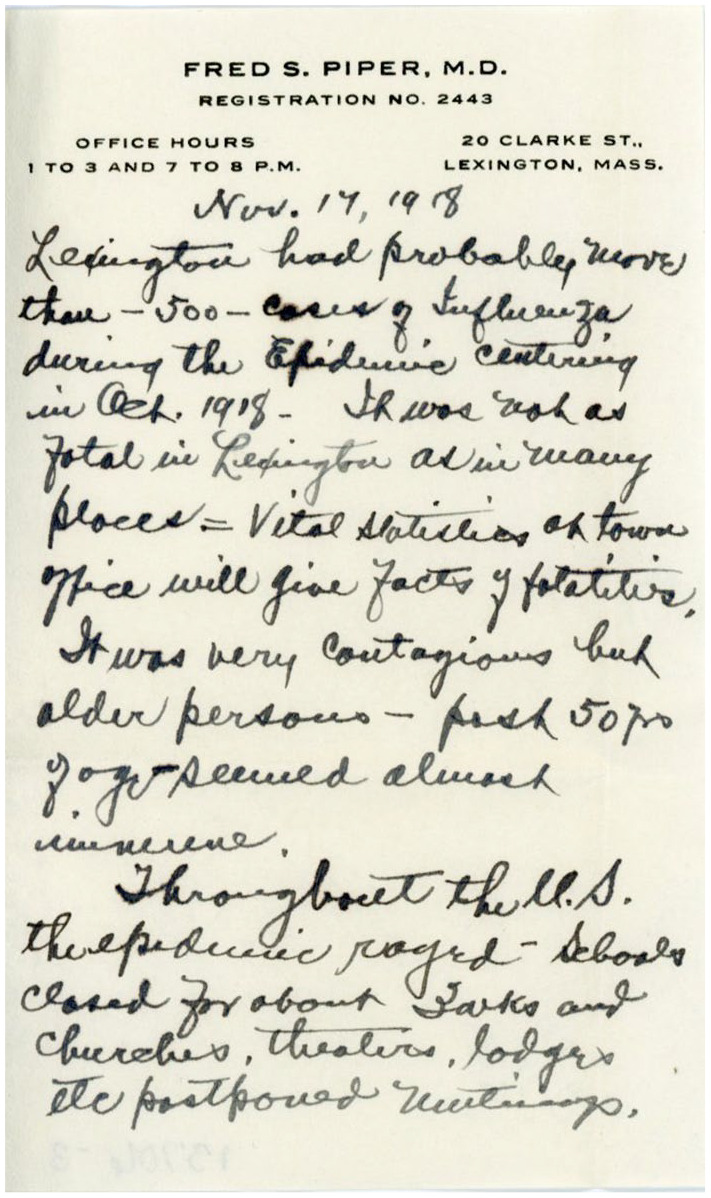
Handwritten record of the influenza epidemic of 1918 in Lexington, Massachusetts documented by Dr. Fred S. Piper on November 17, 1918. This is one of the few records housed in the archives at Lexington Historical Society that details this event in the town’s history. Image courtesy of Lexington Historical Society.

Dr. Piper, a resident of Lexington, Massachusetts, was documenting sudden, drastic modifications to daily life. However, he was not documenting the COVID-19 pandemic of 2020, but rather the influenza epidemic of the early twentieth century. This note from Dr. Piper, penned on November 17, 1918, can be found in the archives at Lexington Historical Society (Figure 1). It exists as one of the surprisingly few primary source records housed within the institution’s collections that details this specific incident and time in Lexington’s history.

At the time of Dr. Piper’s note, he recorded that, “Lexington had probably more than 500 cases of influenza during the epidemic centering in October 1918.”^2^ It is astounding that Lexington Historical Society’s archival collections have so few primary source accounts regarding the 1918 epidemic in their holdings, despite more than 500 cases being recorded in what was, at the time, a small community with a population of approximately 6,000. That is a far higher percentage of residents contracting the flu than contracted COVID-19, and yet we know that in 2020, the lives of every individual in Lexington, a town whose population is now over 30,000, have been greatly altered. One might wonder how Lexington Historical Society, an institution so intensely steeped in the town’s rich history, could have so few contemporaneous, first-hand accounts of such a pervasive experience.

## The Importance of Contemporaneous Collecting

Archivists and historians often find themselves wishing that more primary source information existed regarding a particular era, person, or event. Unfortunately, primary source materials such as diaries, letters, and photographs cannot be created retroactively. Of course, events can be recalled and recorded years later by eyewitnesses and others who lived and experienced them. However, data, records, and stories that have been documented immediately retain a unique and inherent value.

Primary source archival records can fall into one of two categories: those created concurrently with unfolding events or those created after the fact in the form of a memoir, oral history, or the like. At the present time, the entirety of the world is attempting to navigate the complexities of the COVID-19 pandemic and its effects on the whole of society. In this moment, it is evident that we are living through an unmistakably historic installment in human history. A strong argument can be made that this type of event demands to be recorded as it is happening, and it falls to archives and museums to be responsible for collecting these contemporary materials. As time goes on, eyewitness accounts and records will be heavily relied upon to interpret this period.

Historical facts, figures, and statistics are often easy to find or calculate years after an event. How many individuals were infected by the epidemic? How many people died? What were the demographics most strongly affected? Even Dr. Piper did not bother to go into numerical details as he wrote about current events in 1918, simply noting that, “vital statistics at town office will give facts of fatalities.”^3^ Statistics can be discovered and successively analyzed. However, what cannot be easily uncovered is the far richer qualitative data. Unless an individual took the time to take a photograph or to compose their feelings and experiences in a letter or diary, we lose this deeper understanding of the past and must remain content with the numerical data and the subsequent extrapolations that can be made from it.

As historians know, with the passage of time, it is far too easy to lose details. Valuable information, anecdotes, and overall passion in one’s recollections can become less accurate, distorted, or seen through the lens of retrospection. It is imperative that we foster the ability to recognize larger turning points in history so that we can record them contemporaneously; moreover, archivists have a professional responsibility to do so.

## Rapid Response Collecting

It is essential to the accurate and comprehensive recording of history that archivists, and all collections and museum professionals, take action in direct response to current moments of historical importance by implementing new collecting initiatives and, where appropriate, rapid response collecting, which is a newer approach within museums and archives. It was originally pioneered by the Victoria and Albert Museum in London, wherein “objects are collected in response to major moments in history” that pertain to an institution’s collecting mission.^4^ For example, in 2017, the Victoria and Albert Museum acquired a sample of the knitted “pussycat” hats worn by participating protesters in the Women’s March.^5^ This kind of immediate response to current events is the type of collecting program that should be considered more often by cultural institutions.

Archives and history museums have always had the mission of collecting materials for future generations. This is not a new trend. Ancient history museums have made it a priority to gather artifacts and antiquities and carefully curate them in a way as to best interpret life in a long-gone civilization. Archives focusing exclusively on the eighteenth century maintain repositories of painstakingly conserved documents with the hopes that, when pieced together, they can give us a reasonably good sense of colonial life. These examples have strong collecting concentrations and clear missions that they are uniquely qualified to fulfill. It would be inappropriate for them to collect contemporaneously in many cases. Where applicable, however, collecting contemporary material is equally as important as collecting historic objects, yet it is a comparatively deficient movement that has not gained significant traction until somewhat recently.

Whenever archival institutions or museums work to record or preserve a modern-day oral history, or whenever they simply participate in the filling of time capsule, they are inadvertently actively engaging in documenting the present. Such actions often provide invaluable information for the future.^6^ However, even today, “few museums have undertaken a thoughtful, systematic approach to their material documentation of life” in the modern era.^7^

As recently as 1987, L. Thomas Frye of the Oakland Museum wrote an article entitled “Museum Collecting for the Twenty-First Century” in which he addressed this lack of modern collecting. He argued that “increasing recognition of cultural pluralism and a new emphasis on the history of society—the history of ordinary people and everyday life—compel museums to examine their collections for appropriate representative material” of the contemporary era, an era of which many institutions are found deficient.^8^

For many, the routines of daily life seem quite ordinary, and even trials and difficulties faced often do not take on meaning until long after we have lived through them. It is hard to recognize in the moment what will serve to be a key turning point highlighted in history books or what was an experience of certain significance. Rapid response collecting is effective, but how do archivists discern these major moments in history as they are occurring?

## Recording in the Moment

Since it can sometimes be difficult to recognize these historic shifts in the present, archival items, like diaries and letters, chronicling, and personalizing these events as they occur can be critical resources to be drawn on at a later date if desired. Successive documentation, such as memoirs or oral histories, are also valuable, as they are still first-hand reminiscences and therefore can be considered useful primary sources. However, they can sometimes lack the urgency and emotion felt in the moment.

To draw another comparison to the 1918 flu epidemic, we can look to the example of Dr. Isaac Starr, a young physician when the outbreak began who later detailed his experiences in an article titled “Influenza in 1918: Recollections of the Epidemic in Philadelphia,” which was published in an issue of *Annals of Internal Medicine* in 1976. Dr. Starr recalled:

As I look back on those unforgettable medical experiences, I can hardly believe that they took place nearly 60 years ago and that I am one of the few remaining American physicians who served during this great tragedy. . . Our experience in Philadelphia was not unique, and the main features of the clinical picture in 1918 deserve emphasis.^9^

Dr. Starr passed away in 1989, and this incredibly valuable memoir, along with his remembrances and insights, could have been lost forever. However, this is a perfect example of how events can be perceived differently in retrospect. In this passage written in 1976, Dr. Starr is recalling his experiences nearly six decades after his third year of medical school in 1918. While his memories of those distressing days are still quite detailed and comprehensive, he is writing his remembrances with the benefit of the wisdom and knowledge that accompany his accomplished career as a physician. It is entirely possible that those later experiences and achievements color his words. While it is likely that nothing Dr. Starr recounts is factually incorrect, it can nevertheless be contended that his account may have been all the more interesting and insightful had he recorded his experiences as they were occurring, when he was a comparatively inexperienced medical student in the thick of caring for ailing patients with no awareness of the outcome, rather than recording them long after his subsequent successes and professional achievements.

It is not uncommon to recognize the importance of events only after their significance has become apparent, as was the case with Dr. Starr. He shared his story when he recognized that the events of 1918 had gained historic importance, stating, “Recent alarm about the possibility of another epidemic has prompted me to record my experiences in the last one, in the hope that medical attendants will be better prepared for what they might have to face than we were.”^10^ Many archivists and collections personnel have also traditionally waited for this inherent importance to develop. Rather than soliciting donations or accounts as events unfold, they patiently wait for willing donors to reach out when they are prepared to share oral histories or part with what have become treasured objects. As a result of this predilection to collect history passively, the role of the modern archivist must be modified and amended.

## Identifying Value in the Modern and Ordinary

Dr. Starr’s story remains a powerful account of a chaotic period. However, the time has come where historical repositories cannot be passive nor apathetic when it comes to collecting and “cannot be content to wait and accept whatever objects or documents chance happens to place into [their] hands. [They] should take an active, deliberate, analytical approach to the issue of selection and documentation,” else all they are left with are the stories and items that others have chosen and have deemed worthy of preservation.^11^ Unique, attractive, and valuable items tend to survive over the years, and they are therefore easier for cultural institutions to obtain and preserve at a later date. Conversely, memoirs of everyday life and ordinary items lacking in monetary value and beauty tend to be overlooked by museums and historians, and they are consequently forgotten or discarded rather than preserved.^12^

What we choose to preserve versus what we dispose of often says something fundamental about what we value. It is interesting, therefore, to note what our culture, as well as cultures of the past, views as waste, as this can prove enlightening and informative by its very nature. The inherent, underlying value of items is often only determined at a far later date, leading to situations where archaeologists eagerly excavate historic rubbish dumps as though the discarded debris is a treasure trove of social history.^13^ The irony develops when items that were disposed of because they were deemed commonplace and worthless occasionally become some of the most precious of all due to their rarity.

In 2005, Heritage Preservation and Institute of Museum and Library Services (IMLS) partnered to create the Heritage Health Index, which was the “first comprehensive survey ever conducted of the condition and preservation needs of all U.S. collections held in the public trust.” They intended to use their findings as “baseline data that [would] be useful in measuring future preservation efforts”^14^ They correctly identified the need for such preservation assessments for cultural, historical, and scientific collections in the United States as, unlike in the majority of other professions and fields, there had never been an evaluation done regarding collections that would “[track] indicators, [measure] growth, [bench-mark] challenges, and [predict] future trends.”^15^ In a press release regarding this report, then-acting director of IMLS, Mary Chute, sought to clarify why our nation’s collections are of value and worthy of concern and preservation, stating that these items are “vitally important, in part because objects take on unanticipated and surprising meanings over time.”^16^ In other words, what was once assumed to be disposable may one day become invaluable.

Items such as routine correspondence or mundane ephemera are items that we may normally discard. Journals and diaries may not be discarded, but they were meant to document daily life for personal or pragmatic reasons and are typically not created with any intent to preserve them. Yet the beauty and value of these types of documents are that they “turn the past into the present, where ideas and actions can be observed as they develop with all of the doubts and hopes that accompany an unknown conclusion.”^17^ These contemporaneous primary sources provide a glimpse into an intimate, informal, and bolder side of history that is rarely found in secondary source material alone. Archivists must anticipate the need for these qualities in the historic record and fight to preserve these facets of history.^18^

Active collecting initiatives do come with their own sets of challenges, though, many of which were touched on in the Heritage Health Index Report. The accumulated data “points to environmental and storage conditions, emergency planning, staffing, and funding as the aspects of collections stewardship with the greatest needs. If these are not addressed, many collections are at higher risk for damage or loss.”^19^ These are valid concerns, and they are obstacles faced by the majority of collecting institutions. Collecting contemporary items is certainly crucial, but without the ability to actively preserve and manage collections in a way that they can be made accessible to the public, the value is diminished. When institutions agree to accept items into their collection, they have agreed to take on the responsibility and associated costs of processing, housing, and caring for these items. The need to collect, therefore, must be appropriately balanced with existing resources. To this end, it is crucial that archivists put forward strong collections management policies which clearly outline the scope of their collections, as “guidelines on what is not collected are as important as those determining what is collected.”^20^ For the sake of properly maintaining collections and preserving the history they house, archives and museums must recognize the importance of collecting concurrently with modern events while simultaneously being conscious to not overextend themselves or their resources.

## Conclusion: If We Do Not, Who Will?

Dr. Piper of Lexington lived through a monumentally historic event in his community and in the world. Yet, outside of the clerical facts and figures, the vast majority of the intimate details of daily life in a small town during the 1918 flu epidemic are forever lost to us because so few believed they were worth recording and preserving at the time. Let us learn from these errors and recognize that history is not just what happened 200 years ago; history is what happened yesterday.

**Figure 2.**
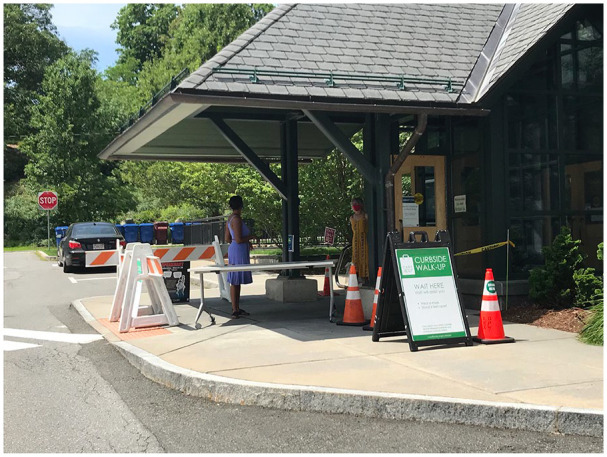
A photograph of masked individuals socially distancing while waiting for curbside pickup at Cary Memorial Library in Lexington, Massachusetts. This is one of the images submitted in 2020 to Lexington Historical Society as part of their recent collecting initiative “What Life was Like in Lexington: The COVID-19 Project.” This project aims to focus on contemporaneous collecting of items which give a sense of everyday life in Lexington during the pandemic. Image courtesy of Lexington Historical Society.

As more conscious efforts are increasingly made to collect more of the provincial vernacular and the contemporary, what we collect today may have no immediate intrinsic value in terms of social distinction, nor may it have astounding monetary worth. Regarding life in the COVID-19 era, a handmade mask sewn by a local to be worn while completing tasks in their everyday life is not innately a priceless piece of folk art. Nor is a family selfie taken on a camera phone during quarantine game night documenting a world-changing historic event. However, as far as its role in preserving a glimpse into daily life during a tumultuous time, what is recorded and collected today may prove to be invaluable to future researchers and historians (Figure 2).

We are living in a historic moment, even if we do not always recognize it as such. As professionals charged with collecting and safeguarding history for our communities and for the future, if we are not attuned to these events, who will ensure that these moments are preserved? To be sure, collecting during a pandemic is met with an abundance of additional impediments to be overcome when interacting with donors and physical collections. Nevertheless, if we do not accept this charge during these most trying of times, then the personal, anecdotal narratives and the intricacies of this history could very well be lost to the future.

## Notes

1. Fred S. Piper, “Record of Influenza in Lexington from Dr. Fred S. Piper,” Box 4, Folder 16, World War I Collection. (Lexington, MA: Lexington Historical Society Collections and Archives, 1918).

2. Ibid.

3. Ibid.

4. Victoria and Albert Museum, “Rapid Response Collecting,”, available at: https://www.vam.ac.uk/blog/tag/rapid-response-collecting (accessed 18 August 2020).

5. *Encyclopædia Britannica*, s.v., “Victoria and Albert Museum,” 2018, available at: https://www.britannica.com/topic/Victoria-and-Albert-Museum#ref1268325 (accessed 18 August 2020).

6. Thomas J. Schlereth, “Collecting Today for Tomorrow,” *Cultural Resources Management*, 16, no. 6 (1993): 8, previously published in *Museum News* 60, no. 4 (March/April 1982).

7. Edith P. Mayo, “Collecting the Twentieth Century,” *History News*, 44, no. 3 (May/June 1989): 30.

8. Thomas L. Frye, “Museum Collecting for the Twenty-First Century,” in *Common Agenda for History Museums: Conference Proceedings* (Nashville: American Association for State and Local History, 1987), 33.

9. Isaac Starr, “Influenza in 1918: Recollections of the Epidemic in Philadelphia,” *Annals of Internal Medicine*, 85, no. 4 (October 1976): 518.

10. Ibid.

11. Schlereth, “Collecting Today,” 9.

12. Edward P. Alexander and Mary Alexander, *Museums in Motion: An Introduction to the History and Functions of Museums*, 2nd ed. (Landham: AltaMira Press, 2008), 194.

13. Schlereth, “Collecting Today,” 9.

14. Heritage Health Index, “A Public Trust at Risk: The Heritage Health Index Report on the State of America’s Collections” (Washington, DC: Heritage Preservation, 2005), 1.

15. Ibid., 3.

16. Mary Chute, “Heritage Preservation Press Release,” *Institute of Museum and Library Services* (December 8, 2005).

17. Lois Marie Fink, “Museum Archives as Resources for Scholarly Research and Institutional Identity,” in *New Museum Theory and Practice: An Introduction*, 2n^d^ ed., ed. Janet Marstine (Malden: Blackwell Publishing, 2006), 293.

18. Ibid., 303.

19. Heritage Health Index, *A Public Trust at Risk*, 1.

20. Frye, “Museum Collecting,” 32.
